# Functional interdependence between septin and actin cytoskeleton

**DOI:** 10.1186/1471-2121-5-43

**Published:** 2004-11-12

**Authors:** Katja Schmidt, Benjamin J Nichols

**Affiliations:** 1 MRC Laboratory of Molecular Biology, Hills Road, Cambridge, CB2 2QH, UK

## Abstract

**Background:**

Septin2 is a member of a highly conserved GTPase family found in fungi and animals. Septins have been implicated in a diversity of cellular processes including cytokinesis, formation of diffusion barriers and vesicle trafficking. Septin2 partially co-localises with actin bundles in mammalian interphase cells and Septin2-filamentmorphology depends upon an intact actin cytoskeleton. How this interaction is regulated is not known. Moreover, evidence that Septin2 is remodelled or redistributed in response to other changes in actin organisation is lacking.

**Results:**

Septin2 filaments are associated with actin fibres, but Septin2 is not associated with actin at the leading edge of moving cells or in ruffles where actin is highly dynamic. Rather, Septin2 is spatially segregated from these active areas and forms O- and C-shaped structures, similar to those previously observed after latrunculin treatment. FRAP experiments showed that all assemblies formed by Septin2 are highly dynamic with a constant exchange of Septin2 in and out of these structures, and that this property is independent of actin. A combination of RNAi experiments and expression of truncated forms of Septin2 showed that Septin2 plays a significant role in stabilising or maintaining actin bundles.

**Conclusion:**

We show that Septin2 can form dynamic structures with differing morphologies in living cells, and that these morphologies are dependent on the functional state of the actin cytoskeleton. Our data provide a link between the different morphological states of Septin2 and functions of Septin2 in actin-dynamics, and are consistent with the model proposed by Kinoshita and colleagues, that Septin2 filaments play a role in stabilisation of actin stress fibres thus preventing actin turnover.

## Background

Septins are a conserved family of GTPases implicated in various cellular processes. Septin-requiring processes include cytokinesis, polarity establishment, cell cycle checkpoints and formation of a diffusion barrier in yeast [1], as well as cytokinesis, vesicle trafficking and exocytosis in mammalian cells [2-4]. In humans, 12 septin genes have been found so far, many of which also undergo alternative splicing generating dozens of polypeptides [5]. Septins can be isolated from cytosol as hetero-polymeric complexes, which have the ability to polymerize and assemble into higher-order structures *in vitro *[6,7]. How the polymerisation is regulated and how such higher order assemblies contribute to septin function *in vivo *is far from clear.

Septin2 (formerly known as Nedd5) is the best-characterised member of the septin family so far. It is ubiquitously expressed and belongs to the acidic subgroup of the septin family consisting of a short N-terminus, a conserved GTPase domain and a coiled coil structure at the C-terminus [8]. Septin2 forms a complex together with Septin6 and Septin7 *in vitro *[9] and also co-localises with these septins *in vivo *[10]. Kinoshita and colleagues showed that Septin2 is required for cytokinesis [11]. Microinjection of an anti-Septin2 antibody interfered with cell division resulting in bi-nucleated cells. How Septin2 functions during cytokinesis is unclear. However, its localisation to the contractile ring and midbody structure in the cleavage furrow during late stages of mitosis is consistent with a functional role of Septin2 in limiting diffusion of membrane proteins across the cleavage furrow [12,13].

In interphase cells Septin2 co-localises with actin bundles, and disruption of the actin cytoskeleton by latrunculin or cytochalasin perturbs Septin2 distribution, inducing curved or circular Septin2-containing assemblies [9,14]. Reduction in Septin2 expression level in cells results in attenuation of actin fibres, implying a functional inter-relationship between actin and Septin2. An *in vitro *bundling assay showed that the interaction between actin bundles and recombinant Septin2/Septin6/Septin7 can be mediated by the bundling protein anillin [4]. Although these *in vitro *results suggest a mechanism to account for the recruitment of Septin2 to the actin contractile ring during cytokinesis, it is unlikely that anillin has the same function in interphase cells. Anillin is sequestered in the nucleus in interphase cells [15] and the functional significance of the co-localisation between actin and Septin2 in this phase of the cell cycle is still elusive. More generally, although artificial perturbation of actin cytoskeleton can cause re-arrangement of Septin2 in cells it is not clear that this phenomenon is ever replicated during normal cell function.

Here we characterise the mutual inter-dependence of the actin cytoskeleton and Septin2 using a range of *in vivo *approaches. Both knock down of Septin2 expression and a specific Septin2 truncation mutant resulted in loss of visible actin fibres or bundles. Expression of dominant-negative mutants of the actin-regulating Rho-GTPases RhoA, Rac1 and CDC42 caused re-organisation of both cortical actin and Septin2. Significantly, in ruffling and migrating cells we observed wholesale redistribution of Septin2 into ring-like structures with morphology and dynamics highly similar to those previously observed upon depolymerisation of actin filaments with latrunculin. We propose that Septin2 is required for actin bundling, and that global re-organisation of the actin cytoskeleton in migrating or ruffling cells triggers concomitant re-organisation of Septin2 into a distinct functional state.

## Results

### Septin2 and actin define interdependent systems

**Figure 1:**
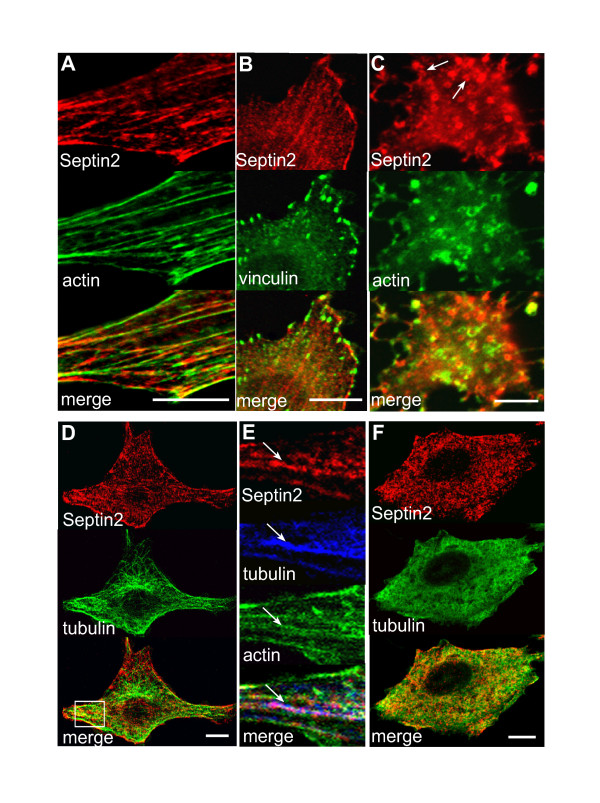
**Septin2 interacts with actin, but not with tubulin. **(A) Immunofluorescence of endogenous Septin2 (red) and actin (green, stained with phallacidin) in interphase NRK cells. Note the typical filamentous-granular organisation of Septin2 and its co-localisation with actin. (B) Septin2 (red) does not colocalise with endogenous vinculin (green), a marker for focal adhesions. (C) Staining of Septin2 (red) and actin (green) after disruption of the actin cytoskeleton. NRK cells were treated with Latrunculin B for 30 min. Septin2 now forms rings. (D) Septin2 (red) and tubulin (green) mainly have distinct distributions in NRK cells. (E) Enlarged image of the boxed region in D. Regions with overlapping Septin2 (red) and tubulin (blue) staining always contain actin (green) as well (arrow). (F) Cells were treated with nocodazole for 30 min. Microtubule organisation (green) is disturbed, but Septin2 (red) distribution is largely unaffected. All images show a single confocal section. Bars, 10 μm.

We sought to understand the functional relationship between actin cytoskeleton and Septin2 *in vivo*. As a first step, indirect immunofluorescence was used to characterise the distribution of these molecules in NRK cells. In interphase cells Septin2 had a filamentous and granular appearance, and co-localised with actin bundles [9]. The interaction with actin bundles, however, was not uniform along the entire length of the actin; Septin2 rather partially decorated the bundle [11]. Septin2 did not co-localise with vinculin, a marker for focal adhesions (Fig. 1B). As previously shown in 3T3 cells [11], disruption of actin filaments upon Latrunculin B treatment resulted in a striking loss of linear Septin2 staining and rearrangement of Septin2 into O- and C-shaped rings (Fig. 1C). These Septin2 rings were sometimes found together with actin-containing particles, but ring-like structures devoid of actin were also visible (arrows in 1C). Thus we could observe the dependence of Septin2 morphology on actin polymerisation described previously [9]. This dependence suggested that septin- and actin-cytoskeletal networks could be functionally inter-dependent. Some members of the septin family also interact with microtubules [16]. For Septin2, however, we were not able to establish such an association (Fig. 1D). In regions of the cell where Septin2 co-localised with tubulin was always actin present as well (Fig. 1E). Nocodazole treatment, which disrupts microtubules, slightly attenuated Septin2 filaments, but did not have a big effect on overall Septin2 organisation (Fig. 1F).

**Figure 2:**
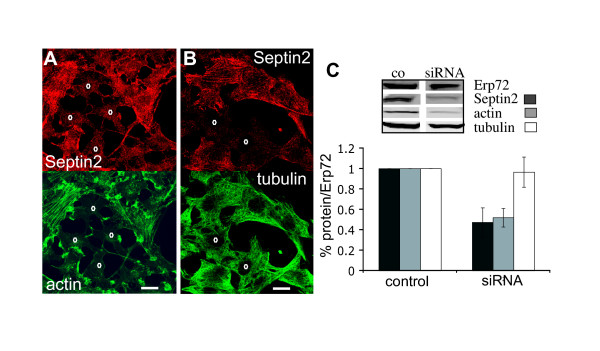
**Knock down of Septin2 expression abolishes actin organisation. **NRK cells were transfected with siRNA targeting Septin2. (A) Immunofluorescence 48 hrs after transfection. Cells with reduced level of Septin2 (red) have no actin bundles (red) (0). (B) Tubulin distribution (green) is unaffected in cells showing a decreased Septin2 staining (red) (0). (C) 48 hrs after transfection with siRNA NRK cells were lysed and immunoblotted for Septin2, actin, tubulin and Erp72. The amounts of Septin2, actin and tubulin were quantified and normalized to the corresponding amount of Erp72 to account for reduction in cell number upon siRNA treatment. co, control; Erp72, Endoplasmic Reticulum Protein, member of the protein disulfide isomerase family. Similar results were obtained with Hela cells. A single confocal section is shown. Bars, 20 μm.

In order to test directly the requirement for Septin2 function in maintaining the actin cytoskeleton, we performed RNAi to knock down Septin2 expression in cells. NRK and Hela cells were transfected separately with two different siRNAs based on the Septin2 sequence. Both siRNAs produced equivalent effects, and control siRNAs produced none of the effects described below. 48 hrs after transfection cell morphology was severely compromised and the overall amount of cells was reduced to 43 ± 11.7% of control levels. 70–90% of the remaining cells showed a decreased Septin2 staining by immunofluorescence. There was no indication of selective arrest of the cell cycle at mitosis or cytokinesis, implying that Septin2 is required for correct cell morphology and adhesion or viability during interphase. Western blot analysis of whole cell homogenate revealed a 60% reduction of Septin2 level (expressed as a percentage of the Endoplasmic Reticulum Protein Erp72, so as to control for reduced cell number; Fig 2C). The most interesting observation was the effect on the actin cytoskeleton. Overall actin level (% of Erp72) was reduced to 45% of control levels (Fig. 2C), and in cells where Septin2 staining was diminished actin staining was markedly reduced as well (Fig. 2A). In fact, actin bundles were no longer present in these cells and the weak residual actin staining was restricted to the cell periphery. In contrast, microtubule organisation was not affected upon knocking down Septin2 levels by siRNA (Fig. 2B) and the amount of tubulin present in cells was unchanged (Fig. 2C).

**Figure 3:**
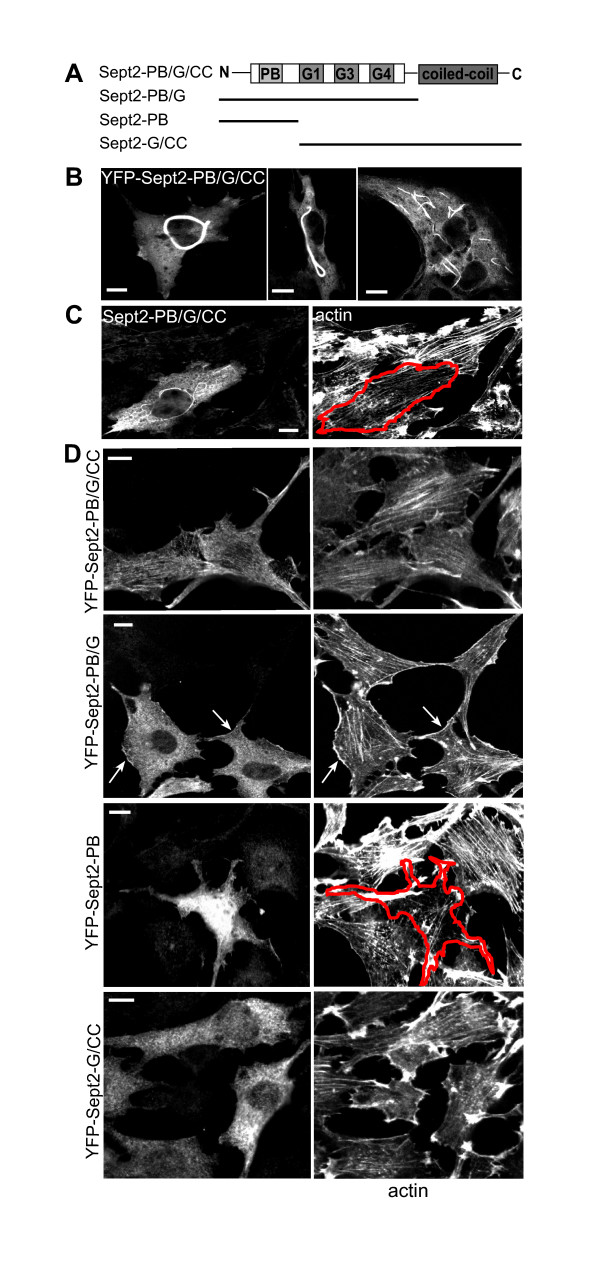
**Characterisation of truncated YFP-Septin2 constructs. **(A) Schematic presentation of full length YFP-Septin2 (YFP-Sept2-PB/G/CC) and truncated YFP-Septin2 constructs. (B) In some cells overexpression of full length YFP-Sept2-PB/G/CC induced the formation of higher-ordered YFP-Septin2 containing-structures with differing morphology (C) Untagged full length Sept2-PB/G/CC is also capable of forming these structures and actin filaments are attenuated in those cells. Full length Sept2-PB/G/CC was detected with anti-Septin2-antibody. (D) Distribution of YFP-Septin2 constructs in NRK cells and their effect on the actin cytoskeleton. Cells were transfected with either full length YFP-Sept2-PB/G/CC or the truncated YFP-Septin2 constructs and stained for actin with phallacidin. Only YFP-Sept2-PB, the construct lacking the GTPase domain and the coiled coil domain affects actin organisation. The distributions of YFP-Sept2-PB/G, the construct without the coiled-coil domain, and YFP-Sept2-G/CC, which lacks the polybasic region, are different to full length YFP-Sept2-PB/G/CC, but there is no effect on actin. Images show a single confocal section. Bars. 10 μm.

The loss of actin fibres in cells with reduced Septin2 expression could either be a consequence of reduced actin protein levels or could reflect a more direct role for Septin2 function in maintaining actin fibres. Given that Septin2 forms complexes with other septins and, potentially, further proteins, we reasoned that in the latter case over-expression of Septin2 might be sufficient to sequester or perturb factors required for actin bundling. Over-expression of full length YFP-Septin2 (YFP-Sept2-PB/G/CC) resulted in the formation of large polymeric structures (Fig. 3B). Untagged full length Septin2 also formed those structures when over-expressed, indicating that their formation was not dependent on the presence of YFP in the fusion protein (Fig. 3C). Induction of these anomalous structures correlated directly with a marked decrease in actin bundles (Fig. 3C). In order to ascertain which region of Septin2 is required for this effect various truncated YFP-Septin2 constructs were produced, containing different domains of the protein [17]. NRK cells transfected with YFP-Septin2-PB/G, a construct containing the N-terminus and the GTPase domain but lacking the coiled-coil domain (Fig. 3A), showed a more or less punctate distribution (Fig. 3D). In comparison to full length YFP-Septin2, no filaments aligned with actin stress fibres were visible, but actin organisation was not affected by over-expression of this construct. In marked contrast, a construct containing only the N-terminal polybasic domain (YFP-Septin2-PB) induced loss of actin bundles, and an apparent reduction in total actin staining to approximately 70% of control levels. YFP-Septin2-PB localized as a cytosolic haze. Staining with Septin2 antibodies revealed that endogenous Septin2 has a normal distribution in YFP-Septin2-PB-expressing cells. A third construct that lacks just the polybasic domain (YFP-Septin2-G/CC) did not affect actin bundles and had a more punctate distribution. Constructs fused to a myc-tag or untagged truncated versions of Septin2 showed the same distributions. Taken together, these experiments show that over-expression of Septin2 is sufficient to ablate actin bundles. However, the large polymeric structures induced by Septin2 over-expression are not themselves required for this effect as a construct containing only the polybasic region of Septin2 is sufficient to induce the same effect on actin bundles whilst having a diffuse cytoplasmic localisation. The finding that the polybasic region plus GTPase domain construct did not have the same effects hints at a regulatory role for the GTPase domain.

**Figure 4:**
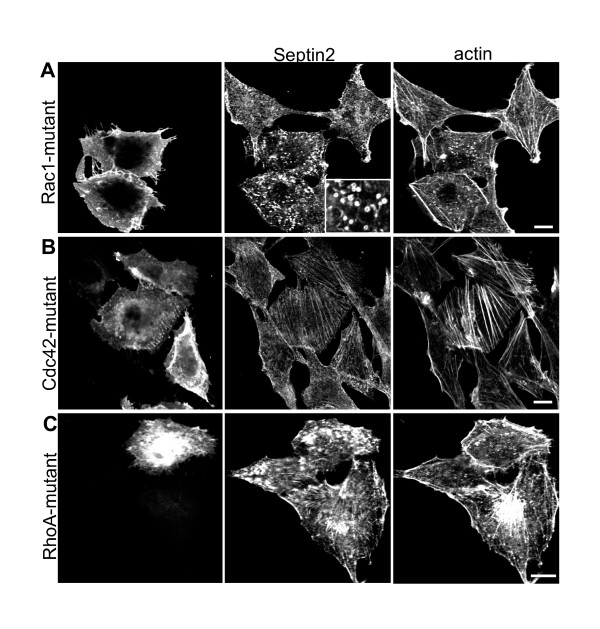
**Effects of Rho-GTPases on actin and Septin2 organisation. **NRK cells were transfected with GDP-locked forms of myc-Rac1 (A), myc-CDC42 (B) or myc-RhoA (C) and stained for Septin2 and actin. GTPases were detected with a monoclonal anti myc-antibody. (A) Cells expressing the myc-Rac1 mutant lack actin bundles and Septin2 organisation is disrupted. Instead of filaments, Septin2 forms rings (inset, inset was taken from another cell transfected with Rac1-mutant). (B) myc-CDC42-GDP induces the formation of thick actin bundles but has only little effect on Septin2 appearance. (C) Transfection with dominant-negativ myc-RhoA causes the detachment of most of the cells. Cells left on the coverslip have diminished actin filaments and disrupted Septin2 organisation. Images are single confocal sections. Bars, 10 μm.

Another way of determining the extent of interdependence between actin and Septin2 is to perturb actin organisation by interfering with regulators of actin organization. Rho GTPases are such regulators and have diverse effects on the actin filament system [18,19]. RhoA, Rac1 and Cdc42 are the best-characterised family members, and each controls the formation of a distinct actin-containing structure. We studied the effects of the GDP-locked form of RhoA, Rac1 and Cdc 42 on Septin2 distribution. NRK cells were transfected with myc-tagged dominant-negative mutants of these GTPases and cells were stained for actin and Septin2. Although all mutants altered the appearance of the actin cytoskeleton and also affected Septin2 organisation, their effects on these two systems were quite diverse (Fig. 4). In cells expressing the GDP-locked form of Rac1 central actin bundles were attenuated or missing and the formation of thick actin bundles in the cell periphery was induced (Fig 4A). Septin2 was no longer associated with these bundles. The filamentous organisation of Septin2 was fragmented resulting in the formation of rings similar to those seen after latrunculin treatment (inset in 4A). The Cdc42-mutant, although increasing the prominence of actin bundles, had only a slight effect on Septin2. Septin2 still exhibited the filamentous-granular appearance but with more pronounced filaments (Fig. 4B). Cells transfected with the dominant-negative mutant of RhoA showed the most severe phenotype. The majority of the cells lost their ability to attach to the surface of the coverslip, which is explained by RhoA having a role in regulation the formation of stress fibres and focal adhesions, necessary prerequisites for proper cell attachment [20]. RhoA-mutant expressing cells that were still left on the coverslip had a disrupted actin and Septin2 organisation, with a reduction in both actin filaments and septin filaments (Fig. 4C). All these experiments again clearly show the mutual dependence and regulation of septin and actin filaments.

### Septin2 and actin have distinct distributions in moving and ruffling cells

**Figure 5:**
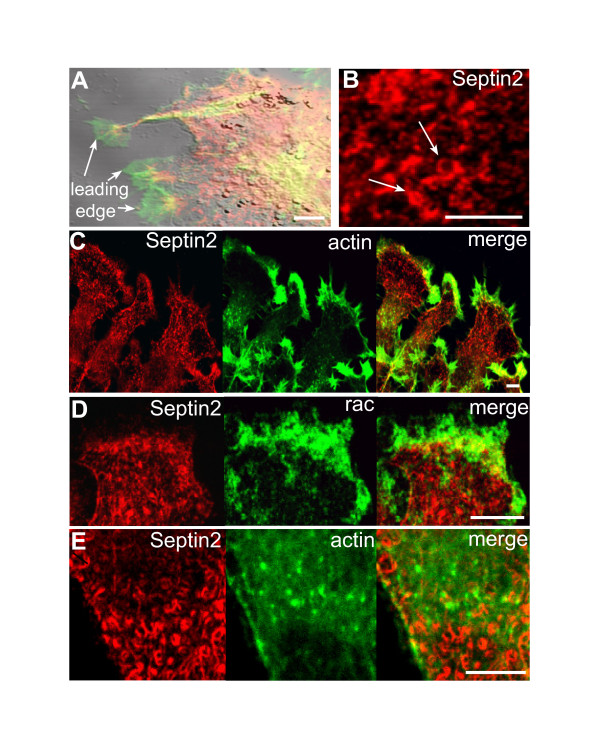
**Septin2 is not associated with actin in moving or ruffling cells. **(A) DIC image of moving NRK cells in an experimentally wounded monolayer. Note the leading edges of the cells contain actin (green), but Septin2 (red) is completely missing. (B) Septin2 forms ring-like structures in the body of moving cells comparable to rings observed after latrunculin treatment (compare Fig. 1C). (C) In ruffling cells growing on lysine-coated coverslips Septin2 (red) is detectable in the cell body, whereas actin (green) is concentrated in ruffles. (D) Ruffles are also positive for endogenous Rac1 (green), but Septin2 (red) is clearly missing. (E) In the cell body of ruffling cells Septin2 (red) forms O- and C-shaped ring-like structures, which are not associated with actin (green, stained with phallacidin). Bars, 5 μm.

Both actin depolymerisation and expression of Rho-family-GTPase mutants are non-physiological perturbations and functional significance of resultant changes in Septin2 distribution was unclear. We therefore sought a more physiological situation where parallel re-organisation of actin and Septin2 could be assayed. In NRK cells migrating into an experimentally wounded monolayer actin is concentrated in the leading-edge lamellipodia and ruffles characteristic of many moving cells (Fig. 5A) [21]. Septin2, however, was not detectable in these ruffling lamellipodia at the leading edge. It was segregated from actin and was localised in the cell body (Fig. 5A). The appearance of Septin2 in the cell body of ruffling cells was distinct to normal cells and very variable. Instead of filaments, Septin2 formed arc-shaped structures, O-and C-shaped rings and circles with diameters ranging from 0.6–1.4 μ (Fig. 5B, arrows), and punctae. The ring-like structures were not at all associated with actin, but were similar to rings observed after disrupting the actin cytoskeleton upon latrunculin treatment (Fig. 1C). Lamellipodial extensions and ruffling can readily be induced in NRK cells by growing on lysine-coated coverslips (Fig. 5C). As one would predict, actin and Rac1, a GTPase known to be involved in formation of lamellipodia, are readily detectable in the ruffles [22]. Septin2 was exclusively localized in the cell body, and was found in O-and C-shaped rings and circles as in migrating cells (Fig. 5D,5E). These results imply that although Septin2 is associated with actin bundles and plays an important role in regulating these structures, it is not associated with actin in situations where actin organisation is more dynamic. Septin2 that does not interact with actin forms variable curled and ring-like structures morphologically highly similar to those induced by actin depolymerisation.

### The filaments and rings formed by Septin2 *in vivo *are highly dynamic

**Figure 6:**
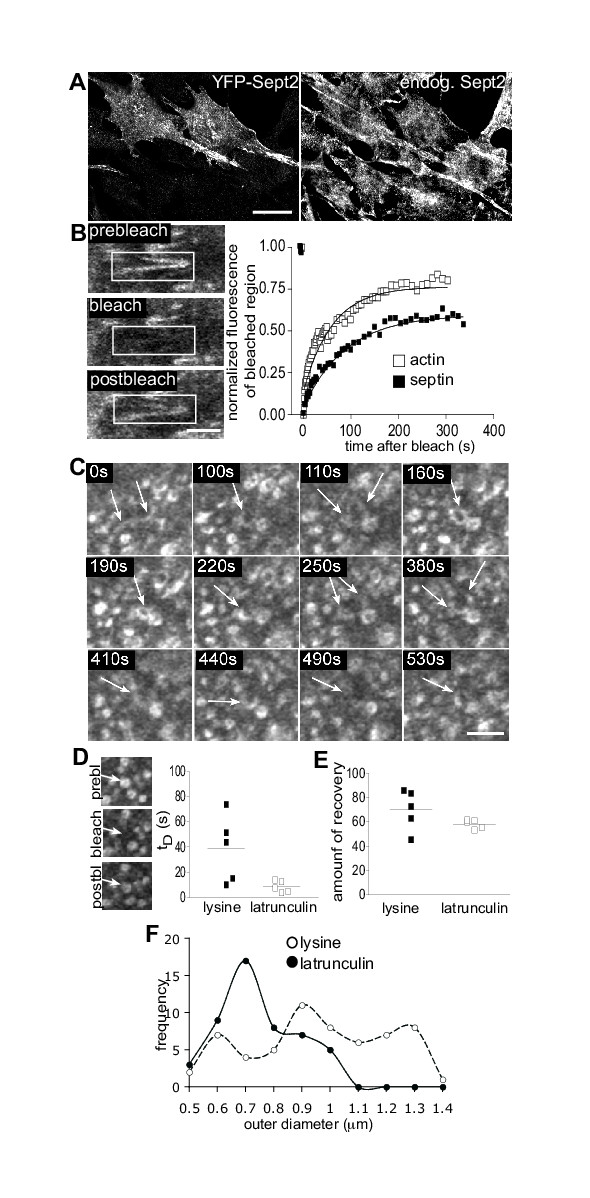
**Septin2 forms highly dynamic filaments and rings. **(A) YFP-Septin2 has the same distribution like endogenous Septin2 and it does not alter the organisation of endogenous Septin2. Bar, 20 μm (B) Photobleach experiment. NRK cells were transfected with either YFP-Septin2 or GFP-actin. Part of the filaments was bleached and recovery monitored over time. The ratio between mean fluorescence intensity of the prebleached box and the mean fluorescence of the whole cell was normalised to the prebleach ratio and expressed as a function of time (Materials and Methods). The graph shows representative recovery curves, which were fitted to a single exponential curve (solid lines) to calculate t_D_s and amount of recovery. n = 5. Images show a single confocal section of YFP-Septin2 filaments in NRK cells. Bar, 4 μm. (C) Images of a time-laps movie of YFP-Septin2 in the cell body of ruffling NRK-cells. Arrows indicate the formation cycle of a ring. Bar, 2 μm. (D,E) Photobleach experiments of YFP-Septin2 rings (also see additional file 1 for lysine/ruffling cells and additional file 2 for latrunculin). NRK cells growing on lysine-coated coverslips to induce ruffles were transfected with YFP-Septin2. Rings formed in the cell body were bleached and recovery monitored over time. For bleaching of Septin2 rings upon latrunculin treatment, normal NRK cells were transfected with YFP-Septin2 and treated with Latrunculin B for 20–25 Min before photobleaching. The data were analysed as in B to calculate t_D_s of recovery (D) and amount of recovery (E). n = 5. Images show a single confocal section of YFP-Septin2 rings in ruffling NRK cells. (F) Quantitative comparison of outer diameters of Septin2 rings in ruffling cells (lysine, empty circles) and upon latrunculin treatment (filled circles). Measurements were done on images of cells transfected with YFP-Septin2.

To gain more insight into Septin2 dynamics we performed fluorescence recovery after photobleaching (FRAP). In a FRAP experiment fluorescent molecules are irreversibly bleached in a small area of the cell (= region of interest, ROI). Subsequent diffusion of surrounding non-bleached molecules into the bleached area leads to recovery and is monitored over time. We used full length YFP-Septin2 for bleaching experiments and investigated the dynamics of GFP-actin in parallel (Clontech). YFP-Septin2 had the same distribution as endogenous Septin2 in interphase and dividing cells and it did not alter the organisation of endogenous Septin2 [12]. First we checked whether Septin2 filaments, which are associated with actin stress fibres in normal NRK cells, are dynamic structures. Photobleaching of a part of the filament showed a recovery of fluorescence over time (Fig. 6B). There was no difference in the dynamics of recovery in the middle or at the end of the Septin2 filament. Although maximal recovery was only about 63% ± 18.74 of the initial fluorescence, the original structure of the Septin2 filament was clearly reformed.

Next we looked at the dynamics of the Septin2 positive ring-like structures formed in the cell body of ruffling cells. Time-lapse movies of these cells revealed a very active behaviour of Septin2 and a relationship between Septin2 filaments and rings. For example, as shown in Fig. 6C starting with an S-shaped filament half of a ring was formed. After 110 s an O-shaped structure with a diameter of 1.45 μm was seen which then further condensed into a smaller ring (Fig 6C, 190 s). The diameter of this ring was 0.7 μm and the morphology was comparable to rings observed after latrunculin treatment (Fig. 1C) and perturbation of actin/Septin2 organisation by Rac1-mutant (Fig. 4A). Instead of remaining in this structure the ring opened again and formed two half rings which were 1.6 μm apart from each other (Fig. 6C, 250 s). The two halves then came together again, this time forming a C-shaped structure (Fig. 6C, 440 s). Many examples of this cycle of opening and closing were observed and sometimes a disappearing and appearing of a ring-like structure was also detected. FRAP experiments of these ring structures in ruffling cells (grown on lysine-coated coverslips) confirmed that constant exchange of YFP-Septin2 into and out of these structures occurs. Halftimes for recovery (ranging from 10 s to 73.8 s) and amount of recovery (45%-86%), however, were very variable (Fig. 6D,6E. Additional files 1 and 2). Sometimes the recovered structure was distinct to the initially bleached structure in terms of position and morphology (see also Additional File 2).

Next we investigated whether Septin2 rings induced by latrunculin have equivalent properties to those seen in ruffling or migrating cells. Septin2 rings formed upon latrunculin treatment also recovered after photobleaching, with comparable dynamics (half-time for recovery was 8.5 s ± 4.5, total amount of recovery 58.3% ± 3.6; Fig. 6D,6E, movie3). Careful morphological comparison of ring structures in ruffling cells and in latrunculin treated cells again revealed considerable similarity. Rings formed upon latrunculin treatment had a slightly more uniform outer diameter (0.74 ± 0.14 μm) but were similar in size to ring-like structures in ruffling cells (0.5 μm-1.4 μm; Fig. 6F). In summary, Septin2 can participate in large-scale macromolecular assemblies with differing morphologies in response to differing physiological situations and remodelling of the actin cytoskeleton. In all instances these assemblies are highly dynamic with constant exchange of Septin2 in and out.

## Discussion

Previous experiments have shown that Septin2 partially co-localises with actin fibres, that actin can affect Septin2 polymerisation *in vitro*, that depletion of Septin2 perturbs the morphology of actin bundles, and that depolymerisation of actin fibres can cause changes in Septin2 morphology. In this study we provide additional direct evidence for the *in vivo *significance of actin and Septin2 interaction. Thus we show for the first time that Septin2 expression is required for maintenance of normal actin protein levels, that over-expression of a truncated version of Septin2 causes loss of visible actin bundling without perturbing the distribution of endogenous Septin2, and that the circular and ring-shaped Septin2 structures induced by actin de-polymerisation are also found in physiological situations where the actin cytoskeleton is radically remodeled.

Partial co-localisation between Septin2 and the tubulin network has also been reported [10]. Our immunofluorescence data, however, argue, that the interaction between Septin2 and microtubules is different from the Septin2-actin interplay and is not crucial for microtubule integrity. Although Septin2 distribution is slightly affected upon nocodazole treatment, this effect is less severe and not comparable with the disruption of Septin2 organisation upon latrunculin treatment (Fig. 1). Moreover, neither knock-down of Septin2 expression upon siRNA nor overexpression of truncated Septin2 constructs affected microtubule organisation (Fig. 2, data not shown). Since in regions of the cell where Septin2 co-localises with tubulin we always find actin as well, we do not think that there is a direct interaction between Septin2 and tubulin, as has been shown for other septins [16,23]. The distinct distribution of Septin9, which is associated with tubulin and is clearly affected upon nocodazole treatment, and Septin2 in dividing cells also suggests that the co-localisation seen between Septin2 and tubulin is not functionally significant [16].

Septin2 and actin distributions, however, are clearly highly interdependent. Overexpression of GDP-locked form of RhoA, Cdc42 and Rac1 highlights this interplay (Fig. 4). At this point we do not know how these GTPases act in this context, whether it is via modulating actin dynamics and/or controlling Septin2 dynamics. It is known that the Cdc42 effectors Borg1, 2 and 3 can bind to Septins *in vitro *and *in vivo *[7,10]. Endogenous Septin6 and Septin7, which form a complex with Septin2, can be immunoprecipitated by an anti-Borg3 antibody and expression of Borg interferes with normal septin distribution. Full-length myc-Borg3 induces the formation of long and thick septin fibres and Cdc42 negatively regulates this effect by inhibiting the binding of Borg3 to septins. These data suggest that the formation of thick Septin2 filaments and actin filaments we see upon expression of the dominant-negative Cdc42 mutant is controlled by a regulatory mechanism, which modulates Septin2 function directly and not only via regulating actin organisation.

In our experiments comparing truncated versions of Septin2 in cells we could show that the GTPase domain of Septin2 is sufficient to prevent the loss of actin bundles induced by expression of the polybasic region alone. The exact role of septin GTPase activity is still unclear [17]. Using recombinant septins, Sheffield and colleagues [7] demonstrated that although pre-assembled Septin6/Septin7/Septin2-filaments show only a slow GTPase activity, GTP-hydrolysis occurs during formation of heterodimers, a process before the assembling of filamentous complexes. Microinjection of non-hydrolyzable GTP (GTPγS) in cells disrupted fibrous distribution of Septin2 suggesting that the fibrous distribution of Septin2 requires GTP-hydrolysis [11]. In contrast, there is no evidence that GTPase-activity is necessary for assembly of septin filaments in curved bundles and ring-like structures in vitro [9]. So far we do not know how Septin2 interacts with actin bundling proteins and how it gets recruited to actin filaments in interphase cells. We do not know whether the reduction in actin expression levels induced by septin2 RNAi is a cause or an effect of the observed loss of actin bundles. Identification of binding partners for Septin2 will be necessary to elucidate the mechanisms underlying the property of Septin2 to stabilise actin bundles.

It has been shown *in vitro *that Septin2 is capable of forming ring-like structures and spirals in an actin-independent fashion [9]. Here, we provide *in vivo *characterisation of this intrinsic property of Septin2. Moving cells and ruffling cells are the first cell systems described so far where the actin-independent distribution of Septin2 in O- and C-shaped rings has been studied in a physiological context, without interfering with cell viability and function. These model systems allow us to draw two firm conclusions: 1. That Septin2 is not associated with actin in regions where highly dynamic actin is not organised in fibres and is being constantly remodeled, and indeed Septin2 is actually efficiently excluded from these regions (e.g. at the leading edge of moving cells and in ruffles): 2. That Septin2 when not associated with actin forms rings and ring-like structures instead of filaments (Figs 5, 6). This is in agreement with *in vitro *data obtained from recombinant septin complexes showing their tendency to self-assemble into rings and spirals [9]. In contrast, however, to these *in vitro *structures, which are highly stable, the *in vivo *assemblies are highly dynamic (movies 1–3). Bleaching experiments clearly showed the constant exchange of Septin2 in and out of the assemblies as well as in and out of actin-dependent Septin2 filaments. The function of actin-independent Septin2 assemblies, however, is still elusive. Since they seem to be freely localised in the cytosol and are not associated with membrane structures (unpublished observations), they might represent storage containers of Septin2. The dynamic behaviour of Septin2 explains how cells can adjust Septin2 function to different needs. The identification of further proteins involved in this process (e.g. GAPs, GEFs, bundling proteins) is necessary for molecular characterisation of the interplay between Septin2 and actin.

## Conclusions

Our data provide a link between the different morphological states of Septin2 and functions of Septin2 in actin-dynamics, and confirm the physiological relevance of the model proposed by Kinoshita and colleagues [9], that Septin2 filaments play a role in stabilisation of actin stress fibres thus preventing actin turnover.

## Methods

### Antibodies and constructs

Anti-Septin2 polyclonal antibody was a gift from M. Kinoshita. Anti-human vinculin monoclonal antibody (clone hVIN-1), anti-c-myc monoclonal antibody (clone 9E10) and anti-α-Tubulin monoclonal antibody (clone DM1a) were all from Sigma. Anti-beta actin monoclonal antibody (AC-15) for immunoblotting was from abcam, BODIPY^®^FL phallacidin and Alexa Fluor ^® ^568 phalloidin for immunofluoresence were obtained from Molecular Probes. Anti-Endoplasmic Reticulum Protein 72 (Anti-Erp72) was from Calbiochem. Myc-constructs of GDP-locked RhoA, Rac1 and CDC42 and the anti-rac monoclonal antibody were kindly provided by H. Mellor. pEGFP-actin was obtained from Clontech. Secondary antibodies used were as follows: Donkey Anti-Rabbit IgG Cy™3 conjugated (Jackson Immuno Research Lab.), Donkey Anti-Mouse IgG Cy™5 conjugated (Jackson Immuno Research Lab.) and Alexa Fluor ^® ^488 goat anti-mouse IgG_1 _(Molecular Probes) for immunofluorescence. IRDye 800 donkey anti-rabbit IgG (Rockland) and Alexa Fluor ^® ^680 goat anti-mouse IgG (Molecular Probes) for immunoblotting.

### Cloning of YFP-Septin2-constructs

To generate full length and truncated YFP-Septin2-constructs, PCRs of Septin2 image clone (Clone Id: 548005, from HGMP, Hinxton/UK) were performed using the following primers: 5'-GCGCTCGAGTGTCTAAGCAACAGCCAAC (sense) and 5'-ATCCCGG GTTACACGTGGTGCCCGAGAGC (antisense) for full length YFP-Septin2-PB/G/CC (nucleotides 1–1081); 5'-GCGCTCGAGTGTCTAAGCAACAGCCAAC (sense) and 5'-CGCCCCGGGTTAGCCTCTCTTGAGTCTCTC (antisense) for YFP-Septin2-PB/G (nucleotides 1–922); 5'-GCGCTCGAGTGTCTAA GCAACAGCCAAC (sense) and 5'-CGCCCCGGGTTACACCATCAGTGTGAACTC (antisense) for YFP-Septin2-PB (nucleotides 1–127); 5'-GCGCTCGAGAGTTCACACTGATGGTGGT (sense) and 5'-ATCCCGGGTTACACGTGGTGCCCGAGAGC (antisense) for YFP-Septin2-G/CC (nucleotides 113–1081). The fragments were cloned in pEYFP-C1 digested with Xho1 and Xma1. To generate untagged full length Septin2 5'-GCGCTCGAGATGTCTAAGCAACA GCCAAC (sense) and 5'-ATCCCGG GTTACACGTGGTGCCCGAGAGC (antisense) were used in the PCR reaction. The fragment was cloned in SNAG4M cut with Xho1 and Xma1. All clones were confirmed by DNA sequencing.

### Cell culture, drug treatment, plasmid transfections, immunofluorescence

NRK cells were grown using standard techniques in DMEM, 10% FCS, 1% Penicillin at 10%CO_2_. To induce the formation of ruffles in NRK cells coverslips were coated with 0.01% poly-D-lysine for 5 min, washed with PBS and air dried before cells were seeded. A wound assay was used to investigate distribution of Septin2 in moving cells. Briefly, NRK cells were grown until confluency and a wound was scratched in the monolayer using a tip. Two hours later cells were fixed and processed for immunofluorescence. To disrupt the actin cytoskeleton or microtubules cells were incubated for 30 min in DMEM containing 150 nM Latrunculin B (Molecular Probes) or 10 μM nocodazole (Sigma), respectively. Plasmid transfections were carried out with Fugene6 (Roche).

For immunofluorescence cells were fixed with 2% Formaldehyd in either PBS or microtubule-stabilisation buffer (0.1 M Pipes, pH 6.9, 2 mM EGTA, 2 mM MgCl_2_, 4% PEG 8000) for 20 min, permeabilised with 0.2% saponin/10%FCS in either PBS or microtubule-stabilisation buffer for 10 min and subsequently incubated with primary and secondary antibodies.

### RNA interference (RNAi) with small interfering RNA (siRNA) and immunoblotting

Two siRNAs with the following sequences: 5'-AAAGGACATGAATAAAGACCA (sense), and 5'-AAGTGAATATTGTGCCTGTCA (sense) were chosen to target nucleotides 939–957 and 521–539 of Septin2, respectively, and were supplied by Dharmacon Research Inc. siRNAs were annealed to make siRNA duplexes according to the manufacture's protocol and transfections were carried out using Oligofectamine (Invitrogen). To check for specificity of knock down we used siRNA duplexes targeting caveolin 1 [24] and rhomboid (gift from A. McQuibban). 48 hrs after transfection cells were processed for immunofluorescence or immunoblotting. For immunoblotting cells were washed with PBS, scratched of in sample buffer (2% SDS, 80 mM Tris/HCl pH 6.8, 10% Glycerin, 0.01% bromphenolblue, 5% β-Mercaptoethanol) and sonicated. The samples were boiled at 80°C for 3 min, centrifuged and the supernatant was loaded onto the gel followed by Western blotting. Immunoreactive bands were detected by the Infrared Imaging System Odyssey (Li-Cor Biosciences).

### Microscopy and photobleaching

All images and movies were obtained using BioRad Radiance and Zeiss LSM510 confocal microscopes equipped with standard filter sets and laser lines for the detection of YFP, Cy2, Alexa Fluor 488, BODIPY, Cy3 and Cy5. Live cell microscopy was carried out at 35°C in imaging medium (DMEM without phenol red, 10%FCS, 50 mM HEPES pH 7.2). Photobleaching experiments were performed with the confocal zoom set to 3 and the confocal pinhole set to 2–4 Airy units. Bleaching of actin or Septin2 filaments was carried out with a 40X 1.3 NA objective lens. A box of interest was bleached using 35 scans with the 488 and 514 laser line at full laser power. For photobleaching of ring structures a 63X 1.4 objective lens was used and a ring of interest was bleached using 25 scans with the 514 laser line at full laser power. Pre- and post-bleach images were monitored at low laser intensity. Fluorescence recovery in the bleached region and the overall fluorescence in the whole cell during the time series were quantified using the Zeiss LSM software. After subtracting the background (= mean fluorescence intensity in the bleached region after bleach) the ratio between mean fluorescence intensity of the bleached region and the mean fluorescence of the whole cell was expressed as a percentage of the pre-bleach ratio of these values. These normalised data were fitted to a single exponential curve using the PRISM software (GraphPad Software Inc., San Diego) to derive amount of recovery and characteristic diffusion time t_D_, which indicates the time at which half of the fluorescence has recovered.

## List of abbreviations used

GFP, green fluorescent protein; YFP, yellow fluorescent protein; siRNA, small interference RNA; RNAi, RNA interference; ERP72, endoplasmic reticulum protein 72; FRAP, fluorescence recovery after photobleaching; ROI, region of interest; GAP, GTPase activating protein; GEF, GTP exchange factor.

## Authors' contributions

KS carried out all the experimental work and drafted the manuscript. BJN conceived of the study, participated in its design and edited the manuscript.

## Supplementary Material

Additional File 1 Photobleaching of YFP-Septin2 ring-like structures formed in the cell body of ruffling cells. Ruffling NRK cells were transfected with YFP-Septin2. The YFP-Septin2 ring in the middle was photobleached and recovery monitored over time. Movie covers 200 s (also see Fig. 6D). Click here for file

Additional File 2 Photobleaching of YFP-Septin2 rings upon latrunculin treatment. NRK cells transfected with YFP-Septin2 were treated with Latrunculin B for 20–25 min before photobleaching. A ring in the upper right part of the cell was photobleached and recovery monitored over time. Note recovery seems to occur from a structure in the background. Movie covers 200 s. Click here for file
